# A Bayesian approach to extracting free-energy profiles from cryo-electron microscopy experiments

**DOI:** 10.1038/s41598-021-92621-1

**Published:** 2021-07-01

**Authors:** Julian Giraldo-Barreto, Sebastian Ortiz, Erik H. Thiede, Karen Palacio-Rodriguez, Bob Carpenter, Alex H. Barnett, Pilar Cossio

**Affiliations:** 1 Biophysics of Tropical Diseases Max Planck Tandem Group, University of Antioquia UdeA, Calle 70 No. 52-21, Medellín, Colombia; 2 Magnetism and Simulation Group, University of Antioquia UdeA, Calle 70 No. 52-21, Medellín, Colombia; 3 Center for Computational Mathematics, Flatiron Institute, New York City, USA; 4 Institut de Minéralogie, de Physique des Matériaux et de Cosmochimie, Sorbonne Université, Paris, France; 5 Department of Theoretical Biophysics, Max Planck Institute of Biophysics, 60438 Frankfurt am Main, Germany

**Keywords:** Cryoelectron microscopy, Molecular modelling, Computational models

## Abstract

Cryo-electron microscopy (cryo-EM) extracts single-particle density projections of individual biomolecules. Although cryo-EM is widely used for 3D reconstruction, due to its single-particle nature it has the potential to provide information about a biomolecule’s conformational variability and underlying free-energy landscape. However, treating cryo-EM as a single-molecule technique is challenging because of the low signal-to-noise ratio (SNR) in individual particles. In this work, we propose the cryo-BIFE method (cryo-EM Bayesian Inference of Free-Energy profiles), which uses a path collective variable to extract free-energy profiles and their uncertainties from cryo-EM images. We test the framework on several synthetic systems where the imaging parameters and conditions were controlled. We found that for realistic cryo-EM environments and relevant biomolecular systems, it is possible to recover the underlying free energy, with the pose accuracy and SNR as crucial determinants. We then use the method to study the conformational transitions of a calcium-activated channel with real cryo-EM particles. Interestingly, we recover not only the most probable conformation (used to generate a high-resolution reconstruction of the calcium-bound state) but also a metastable state that corresponds to the calcium-unbound conformation. As expected for turnover transitions within the same sample, the activation barriers are on the order of $k_BT$. We expect our tool for extracting free-energy profiles from cryo-EM images to enable more complete characterization of the thermodynamic ensemble of biomolecules.

## Introduction

In cryo-electron microscopy (cryo-EM) experiments a biomolecular sample is immersified in vitrified ice. The sample is then irratiated with a low electron dose to take images that correspond to 2D projections of its electron density. Due to advances in electron detection cameras^1^ and improvements in reconstruction algorithms^2^, cryo-EM now enables density maps to be resolved with near atomic resolution^3^, with the highest reported resolution close to 1.22 Å^4,5^. Therefore, cryo-EM now plays a principal role in structural biology for understanding biological systems of a wide range of sizes (from a few kDa to hundreds of MDa)^6^.

The main difference—and advantage—of cryo-EM with respect to X-ray crystallography is that the vitreous ice solution can contain molecules in diverse configurational states. The ultra-fast vitrification process^7^ traps the biomolecules in configurations representative of their temperature before flash-cooling, and the conformational ensemble follows Boltzmann’s distribution. The absence of a single rigid crystalline structure is a great advantage in the study of a biomolecule’s thermodynamic ensemble^6,8,9^. In principle, one can characterize relevant biophysical properties, such as the free-energy landscape, activation barriers, transition states, and transition paths between conformations. This can provide essential clues to biomolecular function^9^.

Several methods have been developed to extract 3D density maps of heterogeneous biomolecules using cryo-EM. These methods can be divided into two types: discrete-state or continuous-state methods. Discrete methods start from a discrete set of reference maps and classify the cryo-EM images according to the map they most resemble. The classified subsets are then optimized iteratively during refinement^10–12^. However, these approaches may be biased towards the initial maps used as templates, and the number of discrete classes must be predetermined^13^. To overcome some of these limitations, continuous-state methods that use principal component analysis (PCA)^14,15^, normal mode analysis^16^ or the covariance matrix^17–19^ have been developed. Combining statistical analysis with optimization algorithms can result in more efficient methods to reconstruct 3D density maps^8,20,21^. However, it is not trivial to determine if the system’s conformational changes are best modeled by a discrete or continuous set of states^13^.

The first studies in which free energies were extracted directly from cryo-EM experiments used particle-classification tools. These studies focused on the prototypical Brownian machine, the ribosome. Fischer et al.^22^ characterized the free-energy landscape of the slow back-translocation process using the number of classified particles for each sub-state ($n_i$, i.e., the occupancy or population of state *i*). The free energy difference with respect to a reference state ($\Delta G$; with population $n_o$) is extracted using the Boltzmann factor, $n_i/n_o = \exp (-\beta \Delta G)$, where $\beta =1/(k_BT)$, $k_B$ is Boltzmann’s constant, and *T* is the temperature. Interestingly, the authors found a relatively flat energy landscape projected along the 30S head versus body rotation at ambient temperature. A similar analysis was also applied to study a pretranslocational mRNA–tRNA sample as a function of the inter-subunit rotation angle^23^. However, these studies are limited by their use of a small number of 3D classes or reliance on time information from the back-translocation process^22^.

An alternative methodology, also initially used to study the ribosome, was developed by Dashti et al.^24^ to extract free energies using the raw cryo-EM particles with diffusion maps. The method selects the images belonging to the same projection direction, then projects the multidimensional free-energy landscape onto a low-dimensional manifold. This method has the advantage that it uses only the raw images without requiring prior 3D classes. Seitz and Frank^25^ use this method together with the POLARIS approach for finding the least action path from 2D energy surfaces. Dashti et al.^26^ also extracted the free-energy surfaces of the ryanodine receptor type 1 (RyR1) associated with the bound–unbound states (with the ATP, caffeine, and Ca$^{2+}$ ligands) using a master equation approach to find the probability of a transition between the two free-energy landscapes. Recently, deep learning methods have provided similar strategies to extract free-energy surfaces^27,28^. We note that replicating these methods might be cumbersome, and the bank of images required is very large. Moreover, the low-dimensional space upon which the particles are projected can be difficult to interpret.

For these reasons, some recent studies have returned to particle-classification schemes for extracting free energies using an increased number of 3D conformations in the classification. Haselbach et al.^29^ studied the dynamics of the Human Spliceosomal $\hbox {B}^{act}$ Complex by performing PCA on the reconstructed 3D volumes. The population of each sub-state along the first two PCA eigenvectors was used to extract the free-energy landscape using the Boltzmann factor. A different study assessed the motion of unbound glutamate dehydrogenase^30^ through a hybrid approach that combined PCA over a molecular dynamics (MD) trajectory (to define the low-dimensional space) with the populations of four cryo-EM maps. The weights of the MD conformations and the relative occupancy of the particles were combined to produce a hybrid free-energy landscape. These methods have the advantage of mapping the free energy onto an easy-to-interpret low-dimensional space. However, PCA assumes that the motions can be modeled in a linear regime, which might not be the case for large conformational changes. Moreover, for highly flexible molecules, generating 3D maps may be challenging.

Free-energy profiling by means of reaction coordinates or collective variables (CV) has been widely used to understand biomolecular processes. CVs reduce the dimensionality of the system by projecting the molecular coordinates onto a low-dimensional, continuous variable (note that PCA is a particular method for constructing CVs). CVs provide a simple and continuous low-dimensional projection of the free-energy landscape of complex multidimensional systems. A good CV should be able to discriminate between key regions of the underlying multidimensional free energy, such as metastable states and transition states. By constructing a free energy profile over the CV and examining features such as barrier heights, practitioners can gain insight into how a reaction takes place and how relevant conformational changes occur. Free energies are commonly extracted by evaluating the CV for each conformation, taking a histogram of the values, and relating the population of each bin to the free energy using the Boltzmann factor. However, approaches based on Bayesian methods also exist^31^. CVs have also been used with enhanced sampling techniques, such as umbrella sampling^32^ or metadynamics^33^, which bias the simulation along the CVs to more efficiently explore the conformational space for extracting the free-energy landscape. Along these lines, several methods^34,35^ have been proposed to extract free energies from MD simulations with CVs that use 3D maps instead of directly using the individual particles.

Inspired by the use of CVs in the MD community^36^, we propose the cryo-BIFE method (cryo-EM Bayesian Inference of Free-Energy profiles), a Bayesian formalism for extracting free-energy profiles and their uncertainties from an ensemble of cryo-EM images. We apply the method to several datasets representing a diverse set of biomolecular systems, using controlled parameters and comparing with known underlying free-energy profiles. We show that under several realistic cryo-EM conditions it is possible to recover the free-energy profile using our methodology. We then apply it with real cryo-EM data to study the transition between the calcium bound/unbound states of a membrane channel. We expect that free-energy profiles from cryo-EM particles will bring new information about the metastable states, barriers, and transition states to help practitioners obtain a more complete thermodynamic characterization of the biomolecular system.

## Theory

### A path collective variable

Consider a biomolecule of *N* atoms. Inspired by Ref.^36^, we will define a collective variable by projecting every possible molecular configuration onto a path in the biomolecule’s configuration space. We will use $x \in {\mathbb {R}}^{3N}$ to denote a particular configuration (conformation). We define the CV in a manner that allows for the extraction of a 1D free-energy profile.

**Figure 1:**
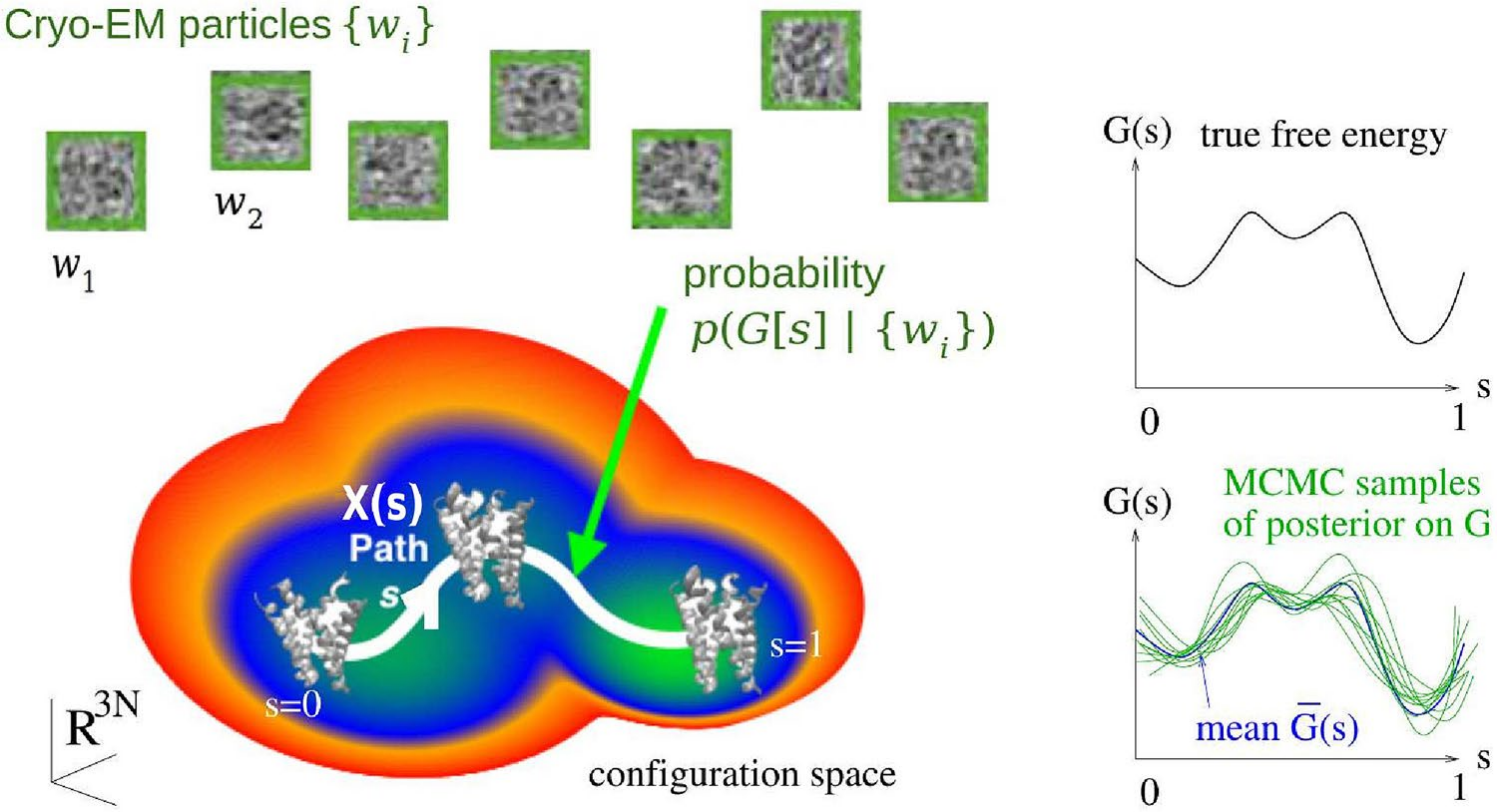
Schematic representation of the path collective variable and Bayesian formalism for cryo-BIFE. The main goal of our methodology is to determine the posterior probability distribution of free-energy profiles *G*(*s*) over a given configuration space path *X*(*s*), given a set of noisy cryo-EM particle (projection) images $w = \{w_i\}$ from $i=1,\ldots ,I$. The green graphs on the right show independent samples drawn from this posterior, and the blue curve their mean. The black curve represents the true free-energy profile. Variation between sampled free energy surfaces arises from a detailed Bayesian model of imaging noise. The path $0\le s \le 1$ is discretized using *M* nodes.

Let a predetermined smooth 1D path *X* in configuration space be parameterized by $0\le s\le 1$, so that $x=X(s)$ is a particular configuration chosen to be on the path. This path should span the relevant conformational changes of the system, and thermal motion should be relatively small in all directions transverse to the path. In Fig. 1, we show a schematic representation of the path *X* (white curve) that connects the relevant metastable states (basins) in the conformational space. At each configuration $x=X(s)$ one sets up transverse coordinates $z\in {\mathbb {R}}^{3N-1}$, so that any configuration *x* in a tubular neighborhood of the path may be written uniquely via a map $x={{\mathcal {X}}}(s,z)$, where $X(s)={{\mathcal {X}}}(s,0)$. This means that inverse functions *S*(*x*) and *Z*(*x*) exist such that ${{\mathcal {X}}}(S(x),Z(x)) = x$ for all *x* in this neighborhood. Our CV is defined by *S*(*x*), i.e. the parameter value *s* of the unique point on the path nearest to a given thermally-accessible configuration *x*. For all points *X*(*s*) on the path, $S(X(s))=s$ extracts their CV parameter.

In practice, one must discretize integrals (e.g., for the Bayesian analysis presented below) over the parameter $0\le s\le 1$. For this we use a simple *M*-node equispaced rule,

1 $$\begin{aligned} \int _{0}^1 f(s) ds \;\approx \; \frac{1}{M} \sum _{m=1}^M f(s_m), \end{aligned}$$

which applies to smooth functions *f*, the parameter nodes being $s_m:=(m-1)/(M-1)$. This defines a discrete set of 3D conformations (which we refer to as nodes) $x_m:=X(s_m)$, that take the system from a starting conformation $x_1$ to a final one $x_M$. Note that *M* is a numerical convergence parameter (the results are expected to converge as $M\rightarrow \infty$), and should be chosen large enough so that conformational changes are small between adjacent nodes. Ideally, the parameterization of the path should also have roughly uniform “speed” $|X'(s)|$, so that discrete conformations $x_m$ are approximately evenly spaced in ${\mathbb {R}}^{3N}$, although satisfying this condition may be challenging in many applications. If the path is well chosen, then the assumption that the cryo-EM images come from conformations near the path is justified by the Laplace approximation in the low-temperature limit, as in path-based algorithms for MD simulations^36,37^.

The CV defined in Ref.^36^ compares 3D conformations (e.g. from an MD trajectory) to the set of nodes belonging to the path *X*. Inspired by this, we develop the cryo-BIFE method, a Bayesian formalism to infer the free-energy profile along the predetermined path, given an ensemble of raw cryo-EM images from the same biomolecule.

### The free-energy profile along the path

Here, we consider the biomolecule at thermal equilibrium. From Boltzmann statistics, the probability density at configuration $x \in {\mathbb {R}}^{3N}$ is given by

2 $$\begin{aligned} \rho (x)=\frac{1}{Z_0}e^{- \beta H(x)}, \end{aligned}$$

where *H*(*x*) is the system’s Hamiltonian (potential energy of conformation *x*), and $Z_0=\int e^{- \beta H(x)} dx$ is the full partition function. We now project this down to the CV. One may choose the map ${{\mathcal {X}}}(s,z)$ so that, at each point on the path, $\frac{\partial x}{\partial z_j}$ for the transverse coordinates $z_j$, $j=1,\dots ,3N-1$, are mutually orthonormal, and orthogonal to the path tangent vector $X'(s)$. Then, near to the path, the Jacobian of the map is the “speed” $|X'(s)|$ (note that $|z|^2$ then matches the squared-distance variable preferred in Ref.^36^). A change of variables gives the marginalized probability density as

3 $$\begin{aligned} \rho (s) = \int \delta \left( S(x)-s \right) \rho (x) dx = \frac{1}{Z_0} |X'(s)| \int e^{-\beta H({{\mathcal {X}}}(s,z))} dz, \quad 0\le s\le 1, \end{aligned}$$

where $\delta$ is the 1D Dirac delta distribution, and in the last step we used Eq. (2) and theJacobian. Since only conformations near to the path are assumed relevant, for simplicity the Jacobian here was approximated as constant with respect to *z*. Note that the final integral in Eq. (3) is a partition function restricted to the “slice” transverse to *X* at *s*. It is then standard to interpret this $\rho (s)$ as the equilibrium density due to an effective 1D free-energy profile (or potential of mean force) *G*(*s*) defined by

4 $$\begin{aligned} \rho _G(s) = \frac{1}{Z_1}e^{-\beta G(s)}, \quad 0\le s\le 1, \end{aligned}$$

a 1D analog of Eq. (2) with $Z_1 = \int _0^1 e^{-\beta G(s)}ds$. Our goal is to infer the function *G* from a large set of 2D cryo-EM images in a statistically rigorous fashion, up to an additive offset. Note that, by Eq. (4), this is equivalent to inferring the population density $\rho _G$.

### cryo-BIFE: a Bayesian approach for extracting the free-energy profile using cryo-EM images

In general, the underlying free energy for a system is unknown. However, in cryo-EM, we have access to a collection of (noisy) raw images $w := \{w_i\}_{i=1}^I$. The model for each image $w_i$ is a noisy unknown projection of the biomolecule with an unknown configuration *x* taken to be independently distributed following Eq. (2). In the CV approach sketched above we restrict this to the 1D configuration path $x=X(s)$, where *s* is a Boltzmann-distributed random variable as in Eq. (4).

For simplicity of notation, we use the symbol *G* to represent the profile, i.e., function *G*(*s*) over $0\le s \le 1$, keeping in mind that in all numerical computations it will be represented by its vector of values at the nodes, $\{G(s_m)\}_{m=1}^M$ (see the Methods). In the Bayesian approach, uncertainty about *G* is encoded by a *posterior* density over the space of functions. Then, by Bayes’ rule,

5 $$\begin{aligned} p\!\left( G | w \right) = \frac{p\!\left( w | G\right) p\!\left( G \right) }{p\!\left( w\right) }, \end{aligned}$$

where $p\!\left( G | w \right)$ is the desired posterior density over free-energy profiles induced by the observed data. $p\!\left( w | G\right)$ is the sampling density (or *likelihood*) of the set of all observed images *w*, assuming a specific free-energy profile function *G*. The term $p\!\left( G \right)$ encodes any prior knowledge about the free-energy profile. In this work, we will impose only a weak-smoothness prior, whose functional form is given in the Methods section. The normalizing constant *p*(*w*), also known as the evidence, will be ignored since it is not needed for inference of *G*. Note that in Eq. (5), and many subsequent formulae, each term is of course conditioned on the path *X*, and thus one could write *p*(*G*|*w*, *X*), etc. However, since *X* is fixed, for notational simplicity we leave this dependence implied.

We assume that the cryo-EM images are conditionally independent given *G,*

6 $$\begin{aligned} p\!\left( w | G\right) = \prod _i p\!\left( w_i | G\right) , \end{aligned}$$

where $p\!\left( w_i | G\right)$ is the sampling density (likelihood) of the single image $w_i$ given *G*.

Our imaging model, encoded by $p(w_i|G)$, may be interpreted as having two steps: first we draw *s* randomly according to $\rho _G$ in Eq. (4), then we draw a noisy image of the 3D molecular configuration $x=X(s)$ according to the full random set of imaging parameters (orientation, translation, noise, etc). Because *s* is an unobserved (a.k.a. latent) variable, the likelihood of an image can be computed by *marginalizing* over *s*,

7 $$\begin{aligned} p\!\left( w_i | G\right) \;=\; \int p(w_i | X(s)) p(s|G) \, {\text {d}}s \;\approx \; \frac{1}{M} \sum _m p(w_i | x_m) p\!\left( s_m | G \right) , \end{aligned}$$

where the second step applies the quadrature, Eq. (1), and our assumption that images come from conformations near the path. The second factor in this sum is, under the Boltzmann assumption, the normalized equilibrium density (4) evaluated at the *m*th parameter node,

8 $$\begin{aligned} p\!\left( s_m | G \right) = \rho _G(s_m) = \frac{1}{Z_1} e^{-\beta G(s_m)}~. \end{aligned}$$

The first factor $p(w_i|x_m)$ in the sum (7) is interpreted as the likelihood function of image $w_i$ conditioned on a known conformation $x_m$. The cryo-EM imaging process is quite well understood, and considerable work has gone into evaluating such likelihoods^10,11,38^. Here, we will use the BioEM formalism from Ref.^39^, which uses a set of numerical marginalizations over all imaging parameters, analogous to (but much larger in scale than) the above one over *s*. See the Methods, and Refs.^39,40^, for details about the BioEM calculations. We note that the present method is not limited to the use of BioEM: any other likelihood formalism (e.g., those used for 3D reconstruction^10^) could be inserted.

Plugging Eqs. (6)–(8) into Bayes’s rule, $p(G|w) \propto p(G) p(w|G)$, and dropping irrelevant normalization factors, the posterior becomes

9 $$\begin{aligned} p\!\left( G | w \right) \;\propto \; p(G) \, \prod _i \; \left[ \; \sum _m p(w_i | x_m) \, \frac{e^{-\beta G(s_m)}}{Z_1} \; \right] . \end{aligned}$$

Given a set of particles, the cryo-BIFE algorithm consists of three main steps: (1) define a path *X* and discretize it with *M* nodes $x_m=X(s_m)$, (2) pre-calculate the BioEM likelihoods $p(w_i|x_m)$ for all nodes $m=1,\dots ,M$, for every image $w_i$, then (3) use a Markov chain Monte Carlo (MCMC) method to *sample* from the posterior, Eq. (9), and from these samples—each a possible profile *G*(*s*)— estimate the expected value of the free-energy profile, $\overline{G}(s)$, and also its uncertainty. Steps (2) and (3) are described in the Methods. Step (1), defining the path, is challenging because it depends on the particular system of interest. In practice, we select a set of conformations $x_m$ that go from one relevant state of the system to another, as is done with the CV from Ref.^36^. In future work, we hope to adapt algorithms from the molecular-simulation community, such as the String method^37,41^ and Nudged Elastic Band^42^, to let us determine optimal path-CVs directly from the cryo-EM data.

In the following, we validate and test cryo-BIFE over a diverse set of systems, from a conformational change along one dimension, using synthetic images, to a membrane channel’s calcium bound/unbound transition, using real cryo-EM data.

## Results

To understand the effects of the physical parameters (e.g., those involved in the image formation process) for recovering free-energy profiles with cryo-BIFE, we designed several control systems where the projections are generated synthetically following the ideas of Ref.^43^. The first system consists of conformations of the Hsp90 chaperone representing a low-dimensional (1D–2D) conformational space. The analysis is then extended to more realistic ensembles from MD simulations. Lastly, we apply cryo-BIFE to experimental cryo-EM data. To this end, we chose raw images of TMEM16F, a membrane channel and lipid scramblase^44^ available at the EMPIAR databank^45^.

### Free-energy profile recovery over controlled datasets

#### Hsp90 chaperone

Hsp90 (a heat shock protein) is a chaperone involved in the folding process of several kinases, transcription factors, and steroid hormone receptors^46^. This protein consists of two chains (A and B, containing 677 residues each) forming a V-like shape. Although Hsp90 is flexible, in the presence of certain ligands (e.g., ATP) its conformational space can be reduced to a few degrees of freedom that go from an open to a closed state of the chains. Following the ideas described in Ref.^43^, we reduced the open-closed dynamics of the Hsp90 into a one (1D) and two (2D) dimensional phase space where both chains are rotated in mutual, normal directions and perpendicular to the axis of symmetry (see the Methods).

#### Free-energy profile recovery for a 1D conformational change

**Figure 2:**
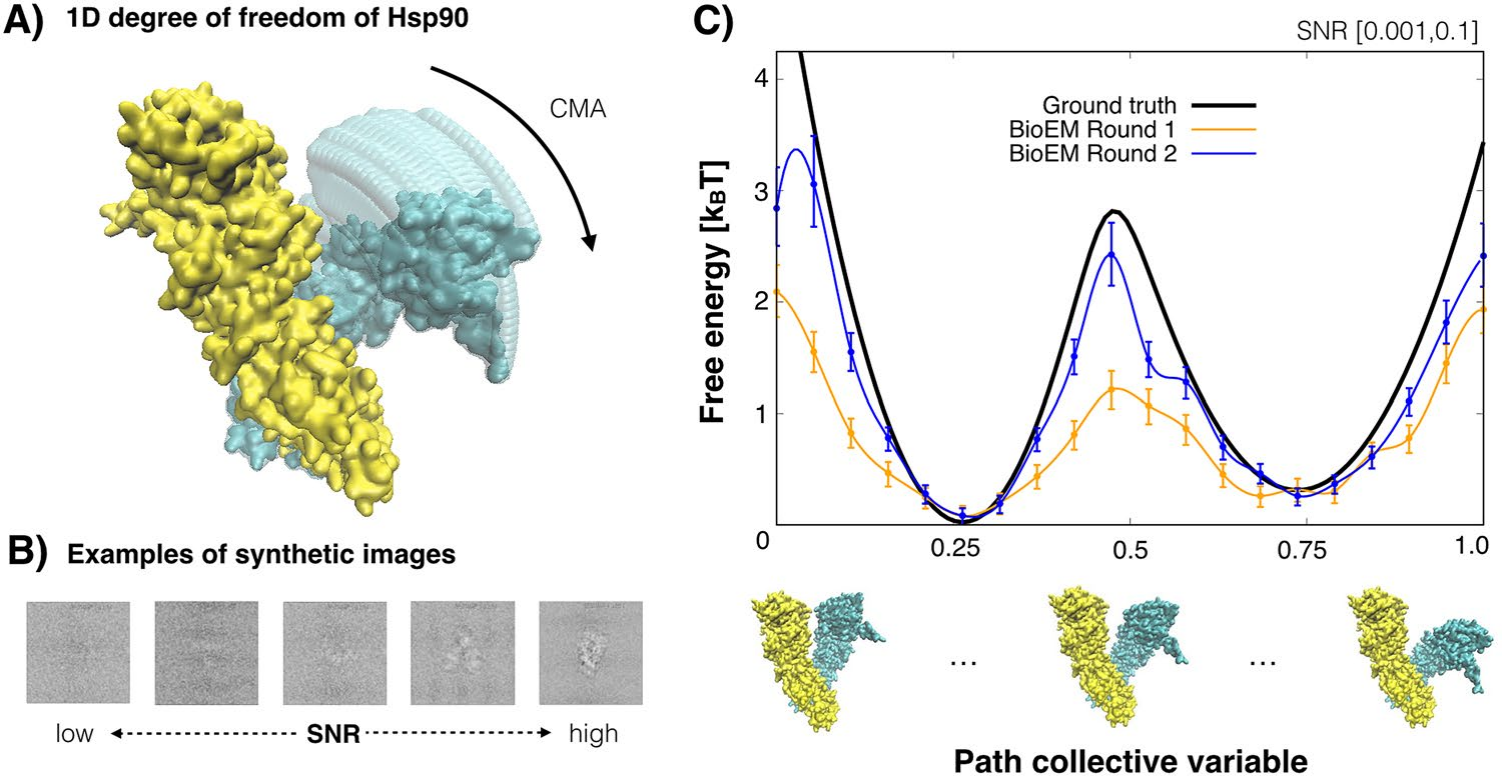
1D analysis of Hsp90. (**A**) Movement of Hsp90 along the single degree of freedom (CMA). The rotation of chain A relative to a fixed chain B. (**B**) Examples of the synthetic images with varying SNR between [0.001, 0.1]. (**C**) Free-energy profiles along the path for the entire set of images recovered from cryo-BIFE. The ground truth free-energy profile is shown in black. The expected free energy profile using cryo-BIFE is shown for BioEM orientation rounds 1 and 2 in orange and blue, respectively. The R-hat test for the MCMC stationarity yielded 1.000 and 1.001 for BioEM round 1 and 2, respectively. The bars show the credible interval at 5% and 95% of the empirical quantile at each node. A cubic spline is used to fit the expected free-energy profile, providing a smooth profile.

In Fig. 2A, we show a 1D conformational change of Hsp90, where chain B is fixed and chain A is rotated from the closed state to the open state (denoted by CMA). We define the path using twenty conformations, equally spaced by $1^\circ$ in the rotation angle. The underlying synthetic free-energy profile (i.e. ground truth) along the path is shown as a black line in Fig. 2C. We generated around 13,300 synthetic images from the predetermined population of the twenty conformations (given by the Boltzmann factor of the ground truth free energy). The synthetic images have a uniform random signal-to-noise-ratio (SNR) $\log _{10}([0.001,0.1])$, defocus [0.5,3] $\upmu$m and orientation angles (see the Methods). Examples of the synthetic particles are shown in Fig. 2B.

To apply cryo-BIFE, we first precalculated the BioEM probabilities for the nodes along the path and all synthetic images for two BioEM rounds of orientation estimation (see the Methods). The MCMC sampling strategy described in the Methods was applied to extract the expected $\overline{G}(s)$ and the credible interval at 5% and 95% of the empirical quantile at each node. Figure 2C, shows the results of $\overline{G}(s)$ using all particles for the first and second BioEM rounds of orientation estimation. Note that the second round was more accurate than the first. This was also reflected in the recovery of the free-energy profile $\overline{G}(s)$, where the second round had a much better performance. This suggests that the pose accuracy of the particles is crucial for extracting an adequate free-energy estimate. The results from BioEM round 2 show that cryo-BIFE was able to recover the free-energy profile for a wide range of SNRs and defocus. Interestingly, the credible intervals widen for higher free-energy values, i.e., near the barrier, where there are fewer particles and the error is expected to be larger. Extracting the credible intervals is the main advantage of using the full posterior in comparison to a *maximum a posteriori* estimation (see Supplementary Fig. 1).

**Figure 3:**
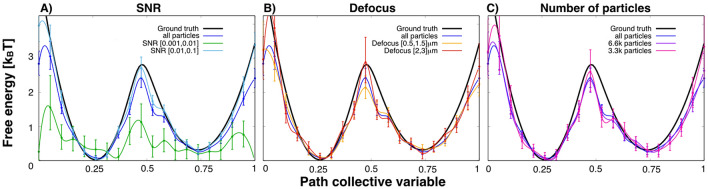
Free-energy profile recovery for different cryo-EM conditions. (**A**) Particles grouped by SNR from [0.01,0.1] (cyan) and from [0.001, 0.01] (green). Each subset contained around 6600 particles. (**B**) Particles grouped by defocus. Sets with small defocus [0.5, 1.5] $\upmu$m (orange) and large defocus [2, 3] $\upmu$m (red). Each subset contained around 5300 particles. (**C**) Particle subsets with a different number of particles: 3300 (pink) and 6600 (purple). For reference, the ground truth and expected free-energy profiles using all particles are shown in black and blue, respectively. The R-hat test for the MCMC yielded values $<1.01$ for all cases. The bars show the credible interval at 5% and 95% of the empirical quantile at each node. The results are for the second BioEM round of orientation estimate.

The performance of the method for different cryo-EM conditions was then studied. In Fig. 3A, the particle set was divided in two: high SNRs from [0.01, 0.1] and low SNRs from [0.001, 0.01], each with an equal number of particles ($\sim 6600$ each). The expected free energy calculated from cryo-BIFE is shown for the high and low SNRs sets (light blue and green, respectively) for the second BioEM orientation round. The expected free energy was also compared to $\overline{G}(s)$ using the entire set (blue line). We observed a poor recovery for the low SNR set [0.001, 0.01] and large errors, whereas the high SNR set behaved well. Interestingly, the free-energy estimate for the entire particle set (SNR [0.001, 0.1]) was slightly worse than for the high SNR set but much better than the low SNR set. The reason for this is that the Bayesian posterior (Eq. (9)) naturally weighs the contribution of each particle and particles with high SNR contribute much more weight to the posterior. If particles with even higher SNR are added (see Supplementary Fig. 2), the free-energy profile recovery is better, and for example, artifacts like the shoulder around $s=0.55$ vanish.

In Fig. 3B, the effects of the defocus by grouping the particles with small defocus [0.5, 1.5] $\upmu m$ (orange line) and large defocus [2, 3] $\upmu$m (red line) were analyzed. The results for the large defocus were slightly better, but these have large errors around the barrier. The number of particles needed to recover the free-energy profile was also studied. In Fig. 3C, the results are shown for sets with 3300 (pink line) and 6600 (purple line) particles. In agreement with previous results for 3D map validation^47^, just a small set of particles ($\ge 3000$) randomly picked from the entire set is able to reproduce the underlying statistics. Contrary to 3D refinement, where large numbers of particles are required, our results indicate that conformational variability can be captured from a small set of particles.

Cryo-BIFE has several advantages over standard particle-classification methods for calculating the populations (or equivalently the free-energy profile). These classification methods treat each particle equally, whereas cryo-BIFE weighs them differently (e.g., depending on their SNR). Moreover, most methods assign each particle to a single node along the path and calculate a histogram over all particles to extract the populations. In Supplementary Fig. 3, this analysis (using the BioEM likelihood) was compared to the cryo-BIFE results for the 1D Hsp90 data with a wide range of SNR [0.001, 0.1]. These results show that cryo-BIFE outperforms standard classification because individual particle-contributions are weighted by the posterior and are not assigned to a single node.

#### 2D conformational change of Hsp90

**Figure 4:**
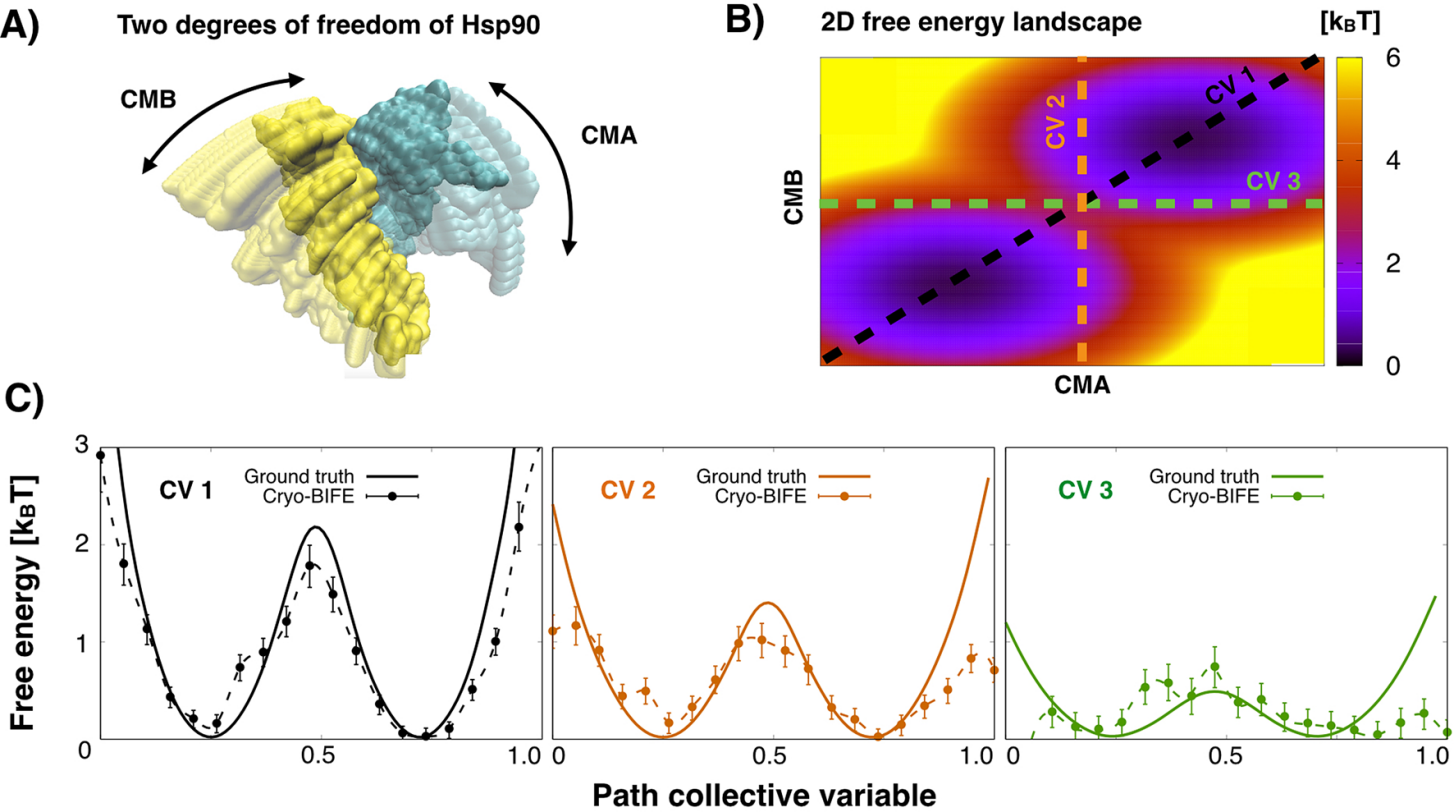
2D analysis of Hsp90. (**A**) Two degrees of freedom of Hsp90 along the CMA and CMB rotation directions (see the Methods). (**B**) Ground truth free-energy surface along CMA and CMB directions. Black (CV1), orange (CV2) and green (CV3) dashed lines show three paths used for the cryo-BIFE analysis. (**C**) The free-energy profiles along these three path CVs, extracted with cryo-BIFE using synthetic particle images (dashed lines), are compared to the ground truth projected profiles (solid lines). The R-hat test for the MCMC yielded values $<1.003$ for all cases. The bars show the credible interval at 5% and 95% of the empirical quantile at each node. The results are for the second BioEM round of orientation estimate.

As described in Ref.^43^, Hsp90 is also characterized by a second degree of freedom; the rotation of chain B relative to the 1D rotation of chain A (see Fig. 4A, and the Methods). A synthetic 2D underlying free-energy surface was generated, shown in Fig. 4B, with an energy barrier of around $2\,k_BT$. Given the imagining conditions in cryo-EM experiments, free-energy barriers around this range are expected. We generated 6800 synthetic particles, using the population given by the Boltzmann factor of ground truth free energy, with SNR [0.01, 0.1], defocus [0.5, 3] $\upmu$m and random orientations in SO(3) (see the Methods).

To study the effects of the path-CV, we defined three paths. The black dashed line (CV1) in Fig. 4B shows a good path-CV that passes along the relevant basins and the transition state of the system. In contrast, the orange and green dashed lines in Fig. 4B (CV2 and CV3, respectively) are able to discriminate between the states (i.e., good order parameters) but are not ideal reaction coordinates because they underestimate the barrier. In Fig. 4C, we compare the expected free-energy profile extracted with cryo-BIFE to the ground truth (given by Eq. (4)) along each path. Relatively good agreement between the underlying profile and the extracted free energy using the cryo-EM images along the three paths was observed. However, using only CV1, the metastable states of the system, the transition state, and true barrier height were recovered. Conversely, using non-ideal CVs, e.g., CV2 and CV3, the barrier can be underestimated. In extreme cases, the identification of the metastable states could also be lost. We note that these are artifacts caused by choosing a poor projection direction, and are not the result of using 2D images. This highlights the importance of choosing an adequate path-CV.

#### Cryo-BIFE over conformational ensembles

**Figure 5:**
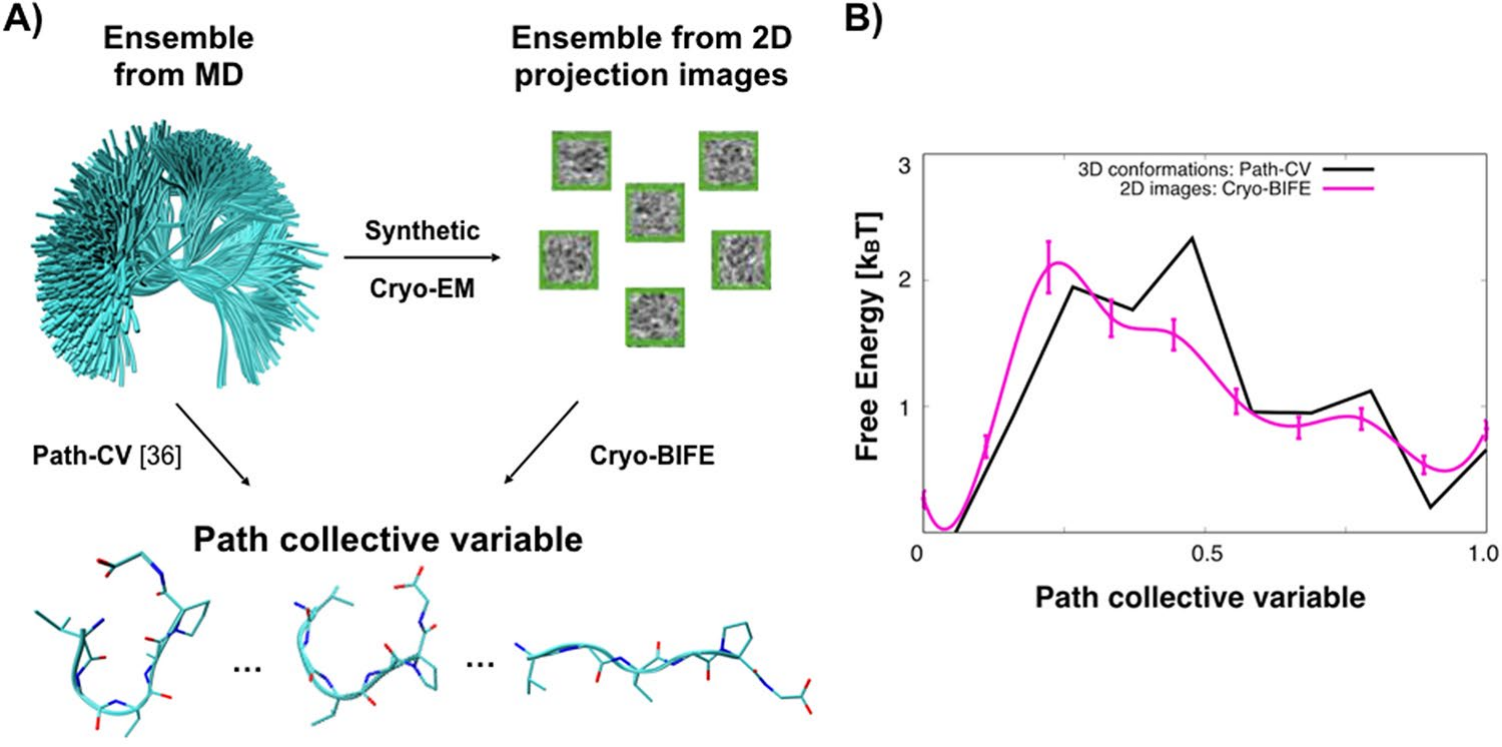
Free-energy profiles from 2D images (cryo-BIFE) or 3D conformations of the VGVAPG hexapeptide. (**A**) The conformational ensemble of the VGVAPG hexapeptide from MD simulations is used to generate synthetic images. The nodes belonging to the path (bottom) are selected with equally spaced end-to-end distances between successive nodes (see the Methods). The path-CV^36^ method compares 3D conformations to the path nodes, whereas cryo-BIFE compares 2D particle images to the same nodes. (**B**) Free-energy profile calculated over the 3D ensemble using the path-CV with RMSD metric [Eq. (8) in Ref.^36^] with $\lambda =50$ Å^ –2^ (black), and the expected free energy $\overline{G}(s)$ extracted using cryo-BIFE with synthetic cryo-EM particles (pink line). The R-hat test for the MCMC yielded values $<1.01$ for all cases. The bars show the credible interval at 5% and 95% of the empirical quantile at each node. See the Methods for details about the path and set of images for each system.

MD simulations of the VGVAPG hexapeptide have been extensively used to test methods, such as Girsanov reweighting^48^. In the Supplementary Information, we present a video showing an example of the hexapeptide MD simulations performed for this work (see the Methods). The peptide has opposite charges at its extremes and exhibits a conformational change between an open state and a closed state. Here, we will compare the free energy extracted from the 3D ensemble to one estimated by cryo-BIFE using 2D particles with the same path (Fig. 5A). The path was created by selecting ten conformations from the MD with equally spaced end-to-end distances between successive nodes (see the Methods). To calculate the free energy from the 3D conformations, we used the path-CV proposed by Branduardi et al.^36^ with the RMSD as a metric. This path-CV was evaluated for each MD conformation, then a histogram was taken and the free energy was calculated via Boltzmann’s factor and the population of each histogram bin. For cryo-BIFE, we used a set of 5688 synthetic images generated from the MD ensemble. The synthetic images had uniformly distributed random SNR, defocus and orientations (see the Methods). Cryo-BIFE was applied to extract the expected $\overline{G}(s)$ along the same path used for the 3D conformations. In Fig. 5B, the free-energy profiles from cryo-BIFE and the path-CV^36^ were compared. The difference is that cryo-BIFE extracts the FE profile from 2D cryo-EM images, whereas the path-CV uses 3D conformations (Fig. 5A).

To investigate whether cryo-BIFE is able to resolve the free-energy profile of membrane proteins with nanodisk belts (as in the cryo-EM experiment), and small conformational changes ($<4$ Å), we attempted to recover a free-energy profile from synthetic images of the semiSWEET transporter generated from MD configurations. Our results are given in the Supplementary Text and Supplementary Figs. 4 and 5. In conjunction with our results on the VGVAPG hexapeptide, they demonstrate that cryo-BIFE is able to recover the free-energy profile from 2D cryo-EM projections for a realistic ensemble.

### Real cryo-EM data: TMEM16F ion channel

**Figure 6:**
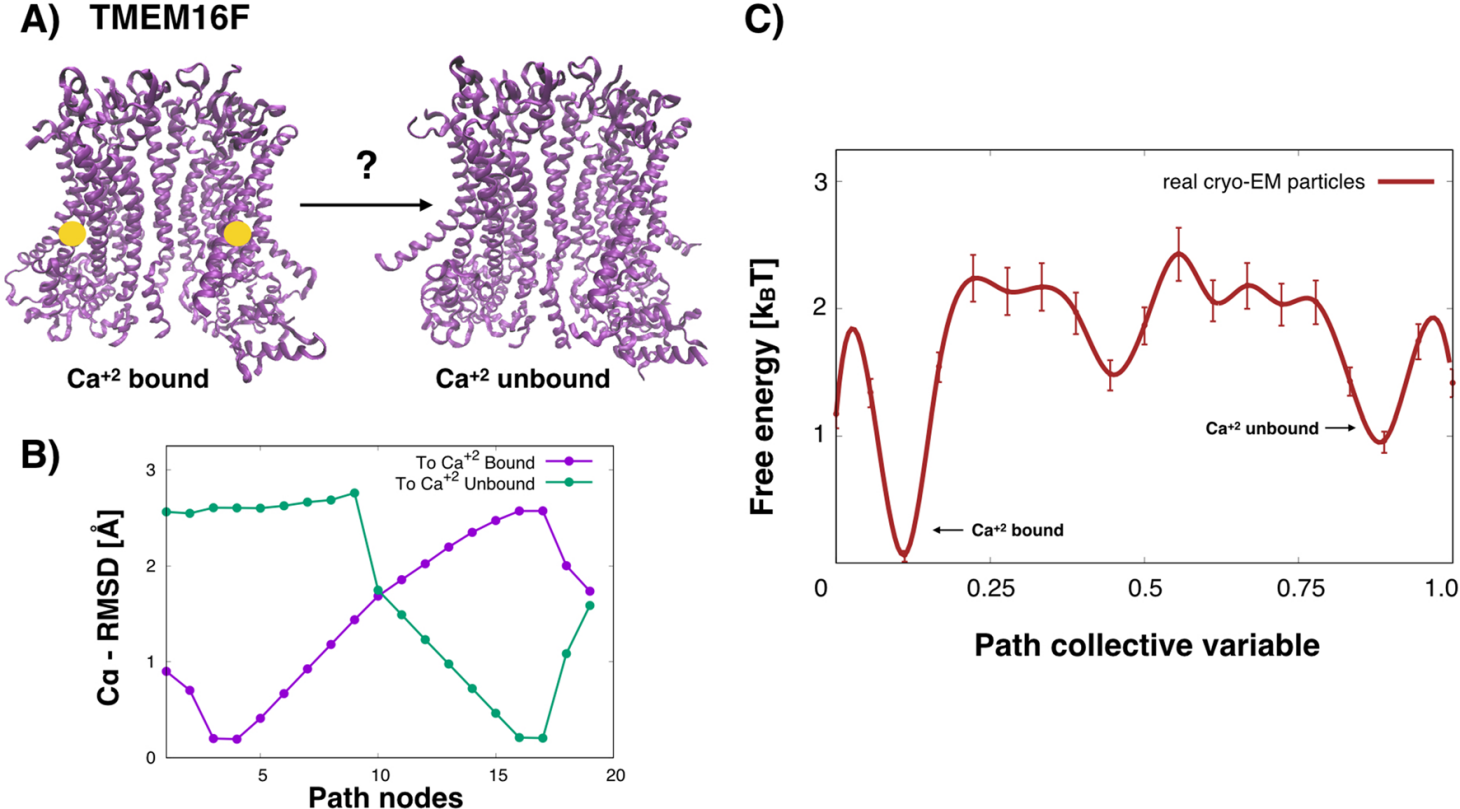
Real cryo-EM data for studying the TMEM16F $\hbox {Ca}^{+2}$—bound/unbound transition with cryo-BIFE. (**A**) $\hbox {Ca}^{+2}$-bound to the $\hbox {Ca}^{+2}$-unbound states of TMEM16F (with PDB codes 6p46 and 6p47, respectively). (**B**) $\hbox {C}_\alpha$ RMSD of the nodes along the path to the $\hbox {Ca}^{+2}$-bound and $\hbox {Ca}^{+2}$-unbound states (purple and green, respectively). (**C**) Free-energy profile extracted along the path CV from real cryo-EM particles from the dataset used to generate the $\hbox {Ca}^{+2}$-bound reconstruction in digitonin^44^ (EMPIAR code 10278). The R-hat test for the MCMC yielded 1.001. The bars show the credible interval at 5% and 95% of the empirical quantile at each node. Arrows point to the free-energy basins corresponding to the $\hbox {Ca}^{+2}$-bound/unbound states.

TMEM16F is a membrane channel and lipid scramblase that is activated by calcium binding. In Ref.^44^, cryo-EM experiments using different $\hbox {Ca}^{+2}$ conditions and membrane/detergent compositions were performed to resolve TMEM16F’s $\hbox {Ca}^{+2}$ bound and unbound states. The cryo-EM particles under different conditions are available at the EMPIAR^45^. In this work, we focus on the EMPIAR dataset with around 1.2 million particles that was used to generate the $\hbox {Ca}^{+2}$-bound state in digitonin (EMPIAR code 10278). Since around 13% of these particles are used to generate the final reconstruction (all other particles are classified out), we wanted to investigate (1) if there could be a small population of the $\hbox {Ca}^{+2}$-unbound state in this set, and (2) if a free-energy profile from the $\hbox {Ca}^{+2}$-bound to the $\hbox {Ca}^{+2}$-unbound states can be extracted. Starting from the PDB structures (Fig. 6A), steered MD simulations were used, which included a lipid membrane and explicit solvent (see the Methods), to generate a path connecting both states. The $\hbox {C}_\alpha$-RMSD of the nodes for both states is shown in Fig. 6B. We randomly selected around 15,000 particles from the entire set, not only those used for the final reconstruction. In Fig. 6C, the free energy along the path using the same cryo-BIFE setup as for the previous systems is shown. It was observed that both the $\hbox {Ca}^{+2}$-bound and the $\hbox {Ca}^{+2}$-unbound states correspond to metastable basins of the system. Because the cryo-EM data set was prepared with $\hbox {Ca}^{+2}$, it is expected that the $\hbox {Ca}^{+2}$-bound state corresponds to the lowest free-energy minimum. However, it is interesting that not all the particles belong to this state, and that the $\hbox {Ca}^{+2}$-unbound state also has metastability. The highest barrier is around 2.2 $k_BT$, consistent with what is expected for turnover conditions in cryo-EM samples. These results show that it is possible to extract a free-energy profile from real cryo-EM particles that agrees with the biophysical setup and expectations of the system.

## Discussion

In this work, we have developed cryo-BIFE, a methodology for extracting free-energy profiles from cryo-EM experiments using a Bayesian approach with a path collective variable. The method was tested and validated over diverse systems covering a range of complexities. Using controlled parameters, we found that the particle orientation accuracy and the SNR are important for adequately recovering the free-energy profile. This work is a proof of principle, demonstrating that under reasonable cryo-EM conditions it is possible to extract free-energy profiles using individual cryo-EM particles.

Primary focus has been given to extracting the *expectation* of the free-energy profile *G*(*s*). However, this method produces (in the form of independent MCMC draws) the full posterior for such profiles, which contains much more information than just an average. In particular it quantifies the degree of certainty with which *G*(*s*) can be extracted given the noise in particle images. Credible intervals can be placed on any function of *G*, such as downstream predictions (reaction rates, etc), simply by evaluating them for all *G* values in a set of MCMC samples.

The cryo-BIFE analysis should be performed on a raw, unbiased cryoEM-particle set. For cryo-BIFE, particles can be picked, polished, and motion corrected. However, 3D-classification methods, which group particles with respect to conformational states, should not be performed before cryo-BIFE because these artificially modify the distribution of conformations. In other words, free-energy profiles extracted from classified-subsets of particles will be biased, and these will not represent the true thermodynamic ensemble.

Here, we have focused on developing, understanding and validating cryo-BIFE for a predetermined path. We have shown that under realistic cryo-EM-imaging conditions the extracted profile coincides with the free-energy profile of the true conformational ensemble along that path. A demanding aspect is how to generate a conformational path for experimental cases. If the metastable states of the system have been resolved using standard cryo-EM 3D classification or from X-ray crystallography, then one could create a path by simply interpolating the maps (or structures) or by using steered MD (as done for the TMEM16F system). If metastable states are not available, then, one could generate conformational paths by directly analyzing the variability of the 2D images, for example, using the covariance matrix or spatialvariational autoencoder (VAE)^49^.

A major challenge remains in determining if the path-CV is optimal. From a thermodynamic perspective, an optimal CV should separate the metastable states of the system, identify the transition states, and activation barriers, corresponding to those of the multidimensional landscape. The lowest free-energy path in the multidimensional space can be considered as an adequate CV. For simulations, several methods have been developed to measure the quality of a CV using transition state theory^50^ or committor analysis^51^, and algorithms exist to find optimal path-CVs^37,41,42^ that can be shown to converge stably ^52^. Recently, additional developments have standardized CV design^53,54^. Nonetheless, a method to determine the optimal path-CV using cryo-EM images is still to be developed. Moreover, for some systems, a single degree of freedom may be insufficient and extending the CV to multiple dimensions would be advantageous.

It is important to note that the temperature plays a crucial role in extracting free energies. In principle, the flash-cooling process^7^ is done rapidly enough that the cryo-EM sample is trapped in the ensemble just before freezing. Consequently, the extracted free-energy profile should be a representation of the system at that temperature. However, freezing takes on the order of $\upmu$s^55^ to complete, so all relaxation processes faster than this timescale are lost. Since vitrification is not instantaneous, cooling might depopulate the barrier and cause the estimated barrier to be artificially large. Other experimental considerations, such as icesheet buckling during vitrification, can cause further perturbations to the observed structural ensemble. It remains to be fully assessed how much the freezing process affects the extracted free energy^56^. On the other hand, to obtain high-resolution reconstructions, it is common to set the system at temperatures below the ambient one for over stabilizing a single state. We hope that these methods to extract free energies will motivate the field to measure more at ambient temperature, and moreover, use all particles (i.e., without having to discard large percentages).

In summary, extracting free energies from cryo-EM experiments opens the field to the assessment of conformational dynamics from a biophysical perspective. By measuring the populations along relevant degrees of freedom, the results go beyond the discussion of discrete versus continuous, and the biophysical mechanisms are truly revealed. Additional clues to biomolecular function are unraveled by the information of the metastable states (e.g., the size and shape of the free energy basins), of the activation barriers and of the location of the transition states of the system, as is common in single-molecule experiments.

## Methods

### BioEM analysis

The likelihoods $p(w_i | x_m)$ in Eq. (9) were calculated using the BioEM algorithm^39^, as follows. Given an image $w_i$ and a 3D conformation (from a density map or atomic model) $x_m$, BioEM computes the probability density $p(w_i | x_m)$ that $w_i$ is a projection of $x_m$. This probability was calculated by integrating the likelihood function $L(w_i\vert \Theta ,x_m)$ (see the Supplementary Text), weighted by prior probabilities $p(\Theta )$, over all relevant physical parameters $\Theta$ for image formation (rotation angles, displacements, CTF parameters, noise variance, normalization factor and offset^39,40^),

10 $$\begin{aligned} p(w_i | x_m) \propto \int L(w_i\vert \Theta ,x_m)p(\Theta ) d\Theta . \end{aligned}$$

The integrals over the noise variance, offset and normalization were performed analytically, and all others were computed numerically, as described in Ref.^40^. The prior densities of the orientation angles and the displacements were taken to be uniform over the integration interval. The prior for the CTF defocus parameter was a Gaussian distribution whose center and width depended on the BioEM rounds described below. The normalization constant in Eq. (10) requires some care, since for Bayes’ rule, hence Eq. (9), to be correct, the likelihood $p(w_i|x_m)$ must be normalized over the space of 2D images $w_i$. It suffices that that the normalization factor is merely independent of configuration $x_m$.

The BioEM orientational integral was divided into two stages referred to as Round 1 and Round 2, respectively. In BioEM round 1, $p(w_i | x_m)$ was calculated by integrating over a uniform orientation grid of 36864 quaternions, which was constructed following the method described in Ref.^57^. The BioEM integration ranges and number of grid points for round 1 are presented in the Supplementary Text for each system. In BioEM round 2, a finer quaternion grid of 125 points was created around the ten best orientations (i.e., with the highest probability) selected from BioEM round 1. In total, a 1250 quaternion grid were used for the second BioEM orientation round. For this round, the Gaussian prior for the defocus was centered at the synthetic/experimental value of each particle and its scale was 0.3 $\upmu$m. This procedure is similar to that described in Refs.^47,58^; however, here we calculated BioEM rounds 1 and 2 independently for each node of the path. We used the BioEM code from Ref.^40^ with CPU and GPU acceleration. For one node along with the path and 10000 particles of $128 \times 128$ size, BioEM round 1 takes $\sim$ 6 h on 24 CPU cores + 2 GPUs, and BioEM round 2 takes $\sim$ 3 h on 24 CPU cores.

Recalling Eq. (9), one needs to evaluate Eq. (10) for every image-node pair, i.e., *MI* distinct evaluations. Then, to estimate the free-energy profile, we used the MCMC algorithm described below to draw samples from its posterior, Eq. (9).

### Markov chain Monte Carlo

We used a Markov chain Monte Carlo (MCMC) method to draw a correlated sample of the free-energy profile *G*(*s*) from the posterior defined in Eq. (9). Such a set of samples captures the full posterior in a much more practical fashion than trying to represent it as a function in the high-dimensional space ${\mathbb {R}}^M$. We found that a standard random-walk Metropolis algorithm, sampling the unknown vector of values $\{G(s_m)\}_{m=1}^M$ at the discrete quadrature nodes, was adequate for our needs. Initial values $G^0(s_m)$ were chosen independently and uniformly at random in $[-2,2]$, for each $m=1,\ldots ,M$. Then, each MCMC step $i=1,2,\ldots ,N_{MC}$ comprised the following sub-steps.

- We randomly selected a node $m \in [1,M]$ with uniform probability.
- We randomly displaced the free-energy profile at the selected node $G^i(s_m) = G^{i-1}(s_m)+\delta g$ where $\delta g$ was uniformly randomly chosen in $[-0.5,0.5] k_{B}T$.
- We shifted the free-energy profile so that $\sum _m G^i(s_m)=0$. Note that the particular choice of shift here is irrelevant.
- We evaluated the posterior in Eq. (9) using the samples $G^i(s_m)$ of this free energy, and the pre-calculated values of $\log (p(w_i | x_m))$ (described above by Eq. (10)) for all images and all nodes $m=1,\dots ,M$. For the prior in Eq. (9), we used $p(G)=\int \lambda e^{-\lambda \mathcal {G}} d\lambda =1/\mathcal {G}^2$, where $\mathcal {G}=\sum _{m=1}^{M-1}(G(s_{m+1})-G(s_{m}))^2$, which is a standard normal prior on the discrete differences, marginalized over the precision parameter $\lambda$.
- From this, the log-acceptance probability of the proposal was computed (here we omit *s* for notational simplicity, so that *G* may be thought of as a vector in ${\mathbb {R}}^M$):
  11 $$\begin{aligned} A (G^{i}, G^{i-1}) \; := \; \log \left( p(G^{i} | w )\right) - \log \left( p(G^{i-1} | w )\right) , \end{aligned}$$
- We chose a uniform random number $u \in [0,1]$. Then, if $\log (u) \le A (G^{i}, G^{i-1})$, the move was accepted, otherwise it was rejected (in which case $G^i =G^{i-1}$).

This procedure was iterated well beyond the time by which the distribution over samples has reached stationarity. For the systems analyzed in this work, we ran $R=8$ independent MCMC chains each with a total of $N_{MC}$ = 200,000 steps. The expected value of the free energy at each node was calculated using all samples $i=1,\ldots ,R\, N_{MC}$, that is,

12 $$\begin{aligned} {\overline{G}}(s_m) = \frac{1}{R\, N_{MC}} \sum _{i} G^i(s_m). \end{aligned}$$

Finally, since it is assumed that the nodes adequately discretize a continuous path, to recover a continuous function $\overline{G}(s)$, we fitted a cubic spline through the values $\{{\overline{G}}(s_m)\}_{m=1}^M$ with knots being the nodes $s_m$. Because only free-energy differences are relevant, we shifted ${\overline{G}}$ such that its minimum was zero. The credible interval for each node was calculated at 5% and 95% of the resulting empirical distribution. We performed the R-hat diagnostic^59^, which compares the inter-chain variance to the variance within each chain to monitor convergence of the MCMC using the arviz package^60^. R-hat values $\le 1.1$ indicate convergence of the sampling.

The MCMC code was written in Python3.5. It was optimized with the Numba compiler, taking approximately 2 h on 24 CPU cores for *I* = 13,000 particles, $M=20$ nodes, and $R=8$ replicas each with $N_{MC}$ = 200,000 MCMC iterations.

### Synthetic particles

We used a modification of the BioEM program^40^ to generate the synthetic cryo-EM particles following similar ideas to those described in Ref.^43^. Each image was created by coarse-graining the molecular configuration (e.g. one taken from an MD simulation) on the residue level. Each residue was represented as a sphere with a corresponding radius and number of electrons^39^. The contrast transfer function (CTF) was modeled on top of the ideal image given a defocus, amplitude and B-factor (for details see the SI of Ref.^39^). For the synthetic particles, the amplitude was 0.1 and the B-factor was $1$Å. Gaussian noise was added on top of the CTF convoluted image. The standard deviation of the noise was determined (as in Ref.^43^) using the SNR and variance of the image without noise (calculated within a circle of radius 40 pixels centered at the box center). All synthetic images were $128 \times 128$ pixels, however, the pixel size varied for each system.

### Benchmark systems

#### Hsp90 system

The Hsp90 chaperone is a flexible protein involved in several biological processes related to protein folding^46^. When bound to certain ligands, its conformational landscape can be approximated by two relative motions of its chains (A and B)^43^. The Hsp90 dynamics was reduced to a 2D dimensional phase space, where both chains are rotated in mutual normal directions and perpendicular to the axis of symmetry. In this work, we first assessed conformations from just one degree of freedom (1D analysis), and then we assessed images from conformations belonging to the 2D conformational space (2D analysis).

To generate the conformations for the first degree of freedom (1D case), we started from the closed state (PDB ID 2cg9^61^), removed the ATP ligand and residues 1–11 to avoid overlapping crashes. Chain B was fixed and chain A was rotated at $1^\circ$ steps around the center of mass of residues LEU674–ASN677, up to $20^\circ$ from the starting position, generating 20 conformations along this degree of freedom (denominated CMA motion^43^). These 20 conformations were used to define the path for the 1D analysis (Fig. 2A). Along this reaction coordinate, we proposed a synthetic free energy (which determines the population occupancy) given by $\exp (-\beta G_{true}(s))=\exp (-(19s-6)^2/8)+\exp (-(19s-15)^2/18)/3$ for $0\le s \le 1$. This ground truth-free energy is shown as a black solid line in Fig. 2C. Using this synthetic population for the conformations along the path, we generated 13,333 synthetic images of pixel size 2.2 Åwith uniformly distributed random orientations in SO(3), SNR in $\log _{10}[0.001, 0.1]$ and defocus in [0.5, 3] $\upmu$m.

For the 2D conformational landscape, we add a new rotation. Starting from each rotated chain A from the 1D case, residues ILE12-LEU442 of chain B were rotated in $2^\circ$ steps around the center of mass of residues LEU442-LEU443, in the normal direction to the plane generated by the 1D movement of chain A and the axis of symmetry. This normal motion mode was referred to as CMB^43^. In total, 400 conformations were generated corresponding to $20\times 20$ rotations. We proposed a 2D synthetic free energy given by $\exp (-\beta G_{true}(u,v))=\exp (-(u-6)^2/18-(v-6)^2/10)+\exp (-(u-15)^2/18-(v-15)^2/10)$ where* u* is the CMA motion and *v* the CMB motion. This density is characterized by two minima localized at models (6, 6) and (15, 15) separated by a barrier of around $2\,k_BT$. We generated 6800 synthetic images of pixel size 2.2 Åwith uniformly distributed random orientations in SO(3), SNR in $\log _{10}[0.01,0.1]$ and defocus in [0.5, 3] $\upmu$m. For this case, we defined three paths: CV1 is a good reaction coordinate that passes through the minima and transition state following the function $u = v$ (black dashed line Fig. 4B), CV2 has model $u=10$ fixed and *v* varying (orange dashed line Fig. 4B) and CV3 has *u* varying and model $v=10$ fixed (green dashed line Fig. 4B).

#### 3D ensemble of the hexapeptide VGVAPG

We used the conformational ensemble of the hexapeptide VGVAPG from a long all-atom MD simulation in explicit solvent. GROMACS^62^ was used to perform a 230 ns MD simulation. The initial conformation was extracted from the crystal structure of the Ca6 site mutant of Pro-SA-subtilisin^63^ with PBD code 3VHQ (residues 171–176)^48^. The peptide was solvated with a cubic water box, centered at the geometric center of the complex with at least 2.0 nm between any two periodic images. The AMBER99SB-ILDN^64^ force field and TIP3P water model were used^65^. Minimization was done with the steepest descent algorithm and stopped when the maximum force was $\le 1000$ kJ/mol nm. Periodic boundary conditions were used. We performed a 100 ps equilibration in an NVT ensemble using the velocity rescaling thermostat^66^ followed by a 100 ps equilibration in an NPT ensemble using Parrinello-Rahman barostat^67^. The MD production run was performed without restraints, with a time step of 2 fs in an NPT ensemble at 300.15 K and 1 atm. We extracted MD snapshots (or frames) every 40 ps, obtaining 5688 conformations (shown in Supplementary video 1).

We selected ten conformations to create the path such that the nodes covered the relevant conformational changes of the system. To do so, we use the end-to-end distance of the peptide, i.e., the distance between the nitrogen atom of the N-terminus, and the carboxyl carbon of the C-terminus^48^. The path was created by selecting ten conformations from the MD with equally spaced end-to-end distances between successive nodes of 1.8Å. The path is shown at the bottom of Fig. 5A, and it was used both with the path-CV^36^ and cryo-BIFE. The path-CV was calculated using the RMSD between all the MD frames and the ten nodes belonging to the path with parameter $\lambda =50$ Å^–2^ [using Eq. (8) of Ref.^36^]. To calculate the free-energy profile, we computed the value of each CV for all MD conformations, summarized with a histogram (with a number of bins equal to the number of nodes along the path), and then estimated the free energy using the Boltzmann factor and the histogram bin populations.

From each MD conformation, we generated a synthetic image with pixel size of 0.3 Å and with uniformly distributed random orientations in SO(3), SNR in $\log _{10}[0.01,0.1]$ and defocus in [0.1, 1.0] $\upmu$m. Using the 5688 synthetic images and the same ten nodes of the path, we performed the cryo-BIFE analysis.

### TMEM16F: experimental cryo-EM data

#### Cryo-EM particles

The cryo-EM particles of the TMEM16F membrane channel used to generate the calcium bound state^44^ from the EMPIAR dataset^45^ with code EMPIAR-10278 were used. See Ref.^44^, for information about the experimental conditions. The images were recorded with a pixel size of $1.059$Å box size of $256 \times 256$ pixels, with defocus values within the interval $[0.5,2.7]~\upmu m$. For this work, we randomly selected 15,000 images from this Ca$^{+2}$-bound (Digitonin_Ca) set. Note that these images represent the entire set and not only those used for the final reconstruction. Since only 13% of the particles from the EMPIAR-10278 set are used to create the Ca$^{+2}$-bound reconstruction^44^, our hypothesis is that not all imaged particles belong to this state. Our aim was to extract a free-energy profile from the Ca$^{+2}$-bound to the Ca$^{+2}$-unbound states using only the cryo-EM particles from the Ca$^{+2}$-added set.

#### Steered MD for creating the TMEM16F path

To generate the path, we used steered MD simulations from the Ca$^{+2}$-bound to the Ca$^{+2}$-unbound state. The simulations were performed as follows. We started from the Ca$^{+2}$-bound structure (PDB ID 6p46). Since the structure has atoms missing, we added these using the Swiss model webserver^68^. We note that because some residues have to accommodate to fit the missing residues the full atom structure was not identical to the PDB. Starting from the full atom model of 6p46, we added the membrane using CHARMM-GUI^69^, in a 3:1:1 ratio of 1-palmitoyl-2-oleoyl-sn-glycero-3-phosphocholine (POPC), 1-palmitoyl-2-oleoylsn-glycero-3-phosphoethanolamine (POPE), and 1-palmitoyl-2-oleoyl-sn-glycero-3-phospho-l-serine (POPS), respectively. A box size of $16.8076 \times 16.8076 \times 17.2012$ nm was used with periodic boundary conditions and 122923 TIP3P water molecules were inserted. We used the GROMACS program^62^ with the CHARMM36M force field^70^. The temperature was controlled in the simulation with the Berendsen thermostat at 300 K, whereas the pressure was controlled with the Berendsen barostat at 1.0 atm^71^. The energy was then minimized using the steepest descent algorithm and stopped when the maximum force was $\le 1000$ kJ/mol nm. We used the leapfrog algorithm to propagate the equations of motion. The long-range electrostatic interactions are calculated using a PME scheme with a 1.2 nm cutoff. We performed two consecutive equilibrations, of 125 ps each, in an NVT ensemble with a time step of 1 fs. Then, we performed two equilibrations in an NPT ensemble, where the first was of 125 ps and time step of 1 fs, and the last was of 1.5 ns, with a time step of 2 fs. For the equilibration in the NPT ensemble, the pressure coupling was of semi-isotropic type. The backbone atoms of the protein were restrained throughout the equilibration runs.

After the MD equilibration, we performed steered MD simulations^72^ using the GROMACS program^62^ patched with the PLUMED 2.5 library^73^. The first target structure for the steered MD was the Ca$^{+2}$-unbound state (PDB ID 6p47). We used the RMSD of the $\hbox {C}_\alpha$ atoms to steer the dynamics between the initial structure and the target structure. The steering harmonic potential had an initial force constant of 5000 and ending at 260,000 kJ/mol/nm$^2$. We noticed that a threshold of 0.2 Å in RMSD to the Ca$^{+2}$-unbound reference was reached very quickly, in less than 1 ns (Supplementary Fig. 6). A second steered MD simulation was needed to go from the initial system (all-atom system) to the 6p46 PDB structure. This steered MD used the same parameters mentioned before. We also ran two short (1 ns) unbiased MD simulations starting from each state (i.e., closest conformation to PDB 6p47 and 6p46). These trajectories allowed us to build a path from the Ca$^{+2}$-bound to the Ca$^{+2}$-unbound states. We used the $\hbox {C}_\alpha$-RMSD to the Ca$^{+2}$-bound state to select 19 nodes, where successive nodes are as equidistant as possible (see Fig. 6B). To mimic the detergent in the cryo-EM images, we included a membrane nanodisk surrounding each node. It was taken from the lipids from the MD simulations, centered at the center of mass of the protein and of 50 Åradius. The nanodisk was modeled in a coarse-grained manner, similarly to the SemiSWEET transporter (see Supplementary Text and Supplementary Fig. 4).

## Supplementary information

Supplementary Information.

Supplementary Video 1.

## Data Availability

The BioEM code is available at https://github.com/bio-phys/BioEM. For the MCMC Python code please contact the corresponding author.
