# Supplementary material for: A Bayesian approach to extracting free-energy profiles from cryo-electron microscopy experiments
Source: Sci Rep. 2021 Jul 1;11:13657. doi: 10.1038/s41598-021-92621-1 (PMC8249403; doi:10.1038/s41598-021-92621-1)
Supplement: Supplementary file 1 — Supplementary Information. [file 41598_2021_92621_MOESM1_ESM.pdf]

Supplementary Information: A Bayesian approach for extracting free-energy profiles from cryo-electron microscopy experiments

Julian Giraldo-Barreto[1,2,‡], Sebastian Ortiz[1,‡], Erik H. Thiede[3], Karen Palacio-Rodriguez[4], Bob Carpenter[3], Alex H. Barnett[3], and Pilar Cossio[1,5,*]

[1]Biophysics of Tropical Diseases Max Planck Tandem Group, University of Antioquia, Medellín, Colombia
[2]Magnetism and Simulation Group, University of Antioquia, Medellín, Colombia
[3]Center for Computational Mathematics, Flatiron Institute, New York City, United States of America
[4]Sorbonne Université, Institut de Minéralogie, de Physique des Matériaux et de Cosmochimie, Paris, France
[5]Department of Theoretical Biophysics, Max Planck Institute of Biophysics, 60438 Frankfurt am Main, Germany
[*]Corresponding author: pilar.cossio@biophys.mpg.de
[‡]Equal contribution.

ABSTRACT

Supplementary Figures

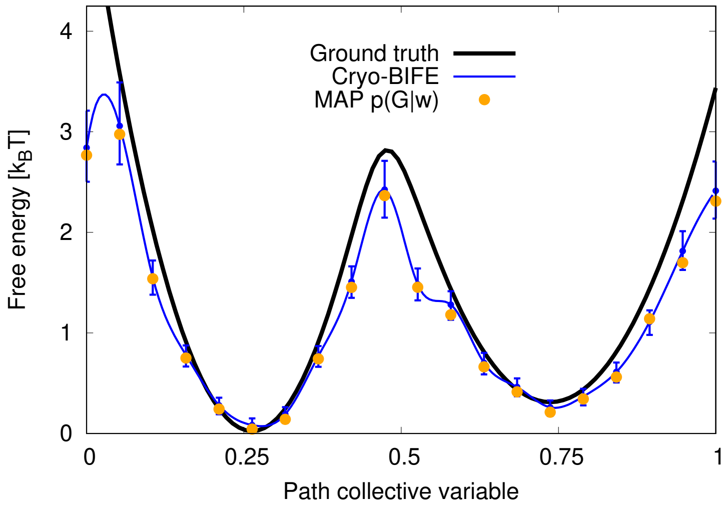

Supplementary Figure 1. Free-energy profiles using the full posterior versus *maximum a posteriori* (MAP) for 1D Hsp90. Free-energy profile recovery using the full posterior from cryo-BIFE MCMC (same as in Main Text Figure 2C blue line) and by extracting the MAP estimation. The images have SNR [0.001,0.1] and the results are for BioEM round 2. In general, we found no major difference between MAP or using the average over the MCMC samples. The main advantage is that with the latter, it is possible to extract the credible intervals, providing information about where the free-energy values are bound.

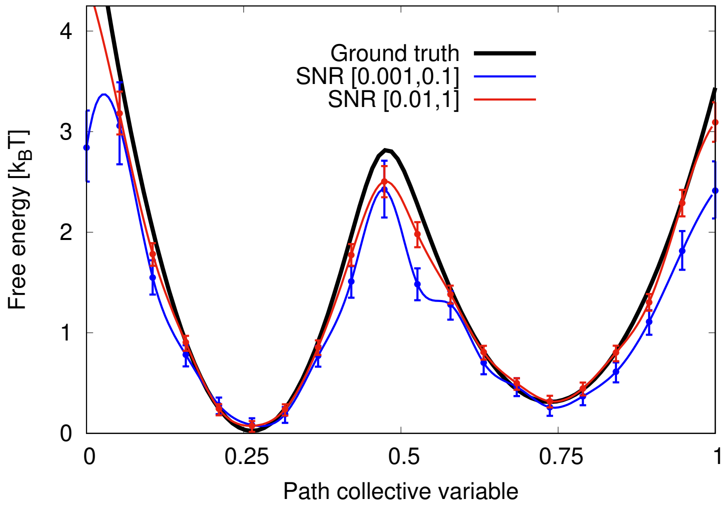

Supplementary Figure 2. Free-energy profiles for Hsp90 particles with a wide range of SNRs. Free-energy profile recovery for particle sets with SNR [0.001,0.1] (same as in Main Text Fig 2C blue line) and with higher SNR [0.01,1] (red line). Each set has 13333 synthetic images with uniformly distributed random orientations in $SO(3)$, SNR and defocus in [0.5,3] μm.

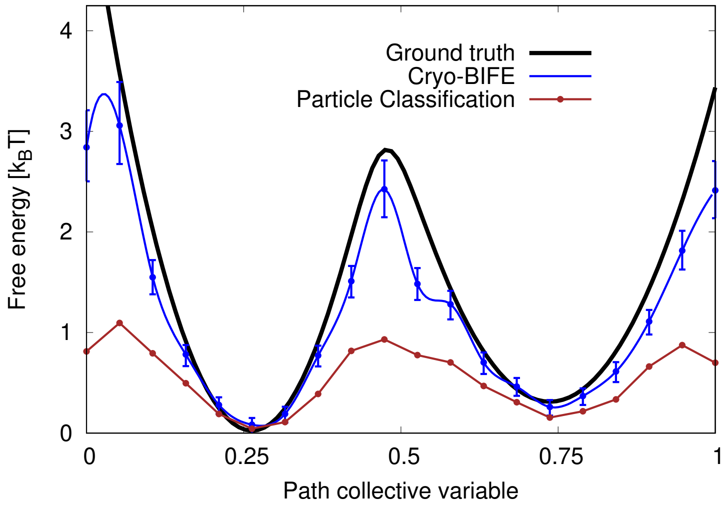

Supplementary Figure 3. Cryo-BIFE versus supervised particle-classification for 1D Hsp90 using images with SNR [0.001,0.1]. Free-energy profile recovery using cryo-BIFE (same as in Main Text Fig 2C blue line) and from particle classification (purple line) by using directly the BioEM likelihood (round 2), assigning each particle to the closest node, calculating a histogram for all particles, and using Boltzman's factor to extract the free energy. Cryo-BIFE outperforms standard classification because individual particle contributions are weighted by the posterior and are not assigned to a single node.

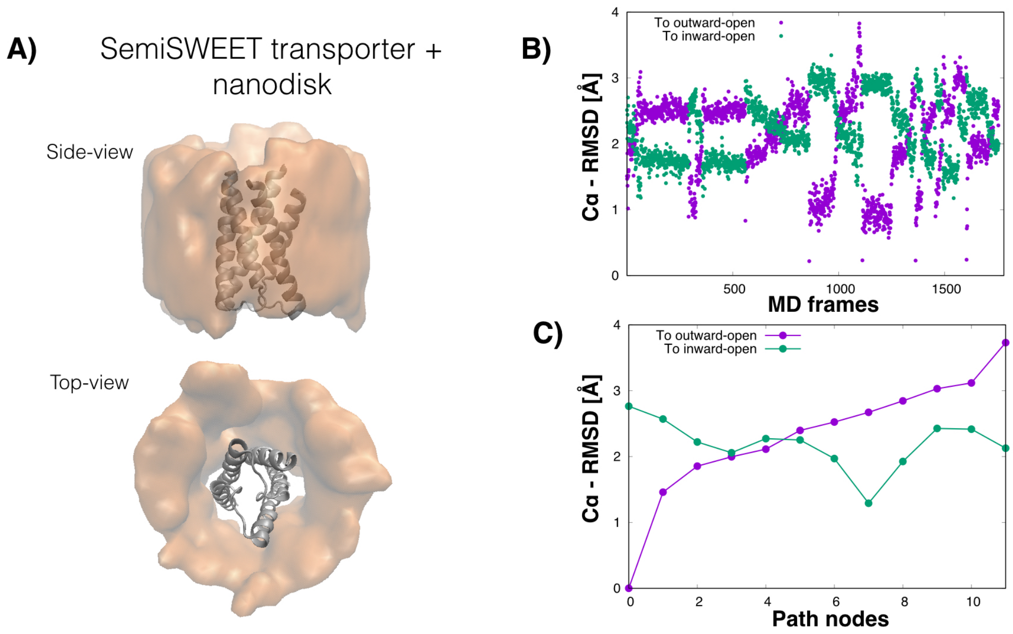

Supplementary Figure 4. Details of the SemiSWEET transporter. A) Example of the coarse-grained nanodisk (orange) used with a SemiSWEET conformation in the generation of the synthetic images and included with the nodes of the path. B) Root mean square deviation (RMSD) of the Cα atoms of the MD conformations and C) of the nodes along the path to the outward-open state (purple) and the inward-open state (green).

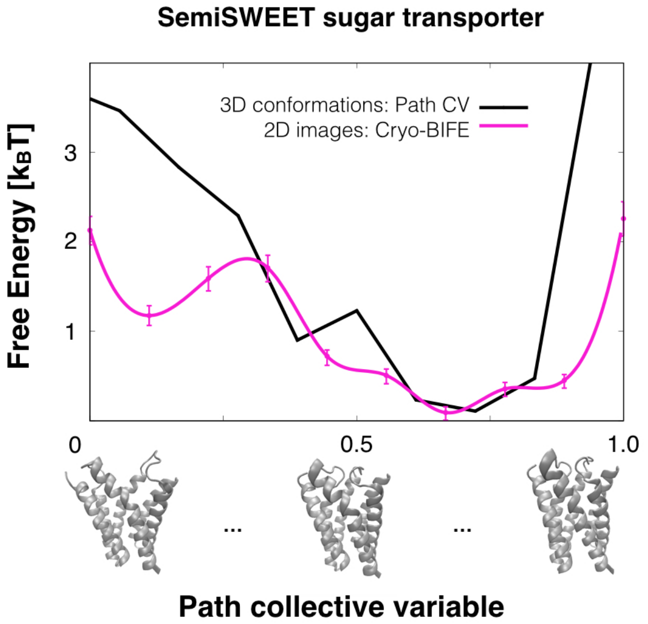

Supplementary Figure 5. Free-energy profiles from 2D images (cryo-BIFE) or 3D conformations for the semiSWEET transporter. Free-energy profile calculated for the 3D ensemble from MD using the path-CV with the RMSD as metric (equation 8 in ref.[1]) and with $\lambda = 50\AA^{-2}$ (black). The expected free energy $\bar{G}(s)$ extracted using cryo-BIFE from synthetic cryo-EM particles (pink line). The R-hat test for the MCMC yielded values < 1.01 for all cases. The bars show the credible interval at 5% and 95% of the empirical quantile at each node.

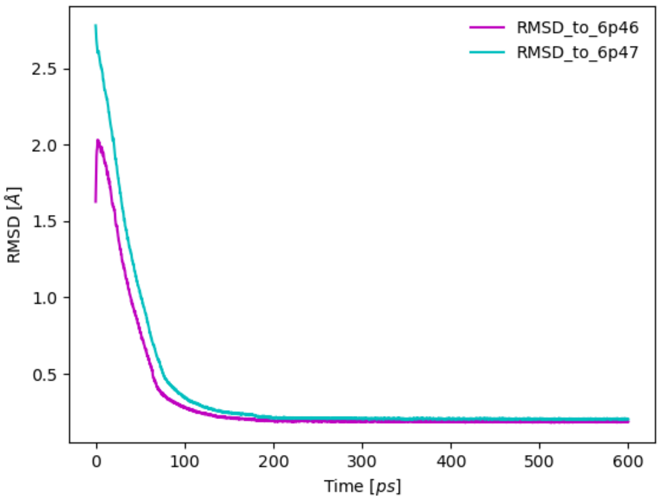

Supplementary Figure 6. RMSD as a function of time in the steered MD simulations of TMEM16F. The cyan and purple curves are the RMSD of the steered MD trajectories towards PDB 6p47 or 6p46, respectively. A subset of conformations from these simulations was selected to represent the path (see Figure 6B Main Text).

Supplementary Text

Cryo-BIFE analysis for MD conformations of the semiSWEET transporter

The semiSWEET transporter is a membrane protein that transports sugar between cell membranes (Supplementary Figure 4A). Several unbiased MD simulations[2] of this transporter were perform starting from the outward-open conformation. Eight trajectories showed a conformational change to the inward-open conformation. The C_α- RMSD of snapshots of these trajectories to both states is shown in Supplementary Figure 4B. We thought it interesting to investigate if cryo-BIFE could resolve the free-energy profile of membrane proteins with nanodisk belts (as in the cryo-EM experiment), and small conformational changes (< 4Å). We used these MD snapshots to generate a synthetic ensemble of semiSWEET conformations, which we used as a reference. We generated 5280 synthetic images from this ensemble having each a nanodisk belt (see below). To define the path, we clustered the MD conformations, and selected successive nodes that had a quasi-equidistant difference in RMSD to the outward-opened state (see below and Supplementary Figure 4C). In Supplementary Figure 5, we compare the free-energy profile from the 2D images to that from the 3D ensemble using Branduardi's path-CV[1]. A relatively good agreement between the profiles around the minimum was found, however, for the high free-energy regions, the agreement was not as good.

Path-CV for semiSWEET

We used the conformations of the SemiSWEET membrane protein from eight unbiased MD trajectories that present a conformational change from the outward-open state to the inward-open state [2]. The RMSD to both states is shown in Supplementary Figure 4B for 1761 snapshots of the trajectories taken every 1ns. We used these conformations as a reference ensemble. The path-CV[1] was used to calculate the reference free-energy profile with the same parameters as described for the VGVAPG hexapeptide (see the Main Text). To select the nodes of the path, we performed a conformational clustering of the 1761 MD snapshots. The GROMACS[3] *g_cluster* tool was used with the RMSD defining the distance between conformations. The clustering was performed using the single-linkage algorithm with an RMSD cutoff of 1.3Å, resulting in 39 clusters. The cluster-center is the conformation with the smallest average distance to all conformations belonging to the cluster. We compared each cluster center to the outward-open and inward-open crystal structures using the RMSD. 12 cluster centers with the quasi-equidistant differences of RMS, between successive nodes, to the outward-open state, were selected (see Supplementary Figure 4C).

Synthetic semiSWEET images

To generate the synthetic images, we used the SemiSWEET conformation together with a nanodisk (*i.e.*, lipid) belt of $25Å$ centered at the center of mass of the protein and extracted from the MD simulation. To coarse-grain the nanodisk and imitate the effects of averaging, the heavy atoms of lipids were modeled with a 3Å radius and 10 electrons (see Supplementary Figure 4A). We generated three images from each MD snapshot (protein and nanodisk) of pixel size $0.7Å$ with uniformly distributed random orientations in $SO(3)$, random SNR $\in \log_{10}[0.01, 0.1]$ and random defocus $\in [0.5, 1.5]\mu m$. A nanodisk belt was also included for each node of the path. We note that for real datasets the lipid nanodisks will have larger uncertainties because of their variable shape and size.

The BioEM likelihood

The BioEM function $L(w_i|\Theta, x_m)$ calculates the likelihood of a 3D conformation x_m to have generated an image w_i given a set of parameters Θ. These nuisance parameters are the projection direction, contrast transfer function (CTF) defocus, CTF amplitude, CTF b-factor, image center displacement, image normalization, offset and standard-deviation of the noise (λ). In essence, the likelihood measures the correlation of the experimental image and a calculated image from x_m using parameters Θ, assuming a Gaussian-noise model. It was defined by[4]

$$L(w_i|\Theta, x_m) = (2\pi\lambda)^{-N_{\text{pix}/2}} e^{-\sum_p (w_i(p) - I^{\text{cal}}(p))^2/(2\lambda^2)}, \quad (1)$$

where p indexes the pixel, and I^{cal} is a calculated image from conformation x_m with a given orientation direction, CTF defocus, CTF amplitud, b-factor, center displacement, image normalization, and offset (see ref.[4] for details about the image-formation process). As mentioned in the Methods, the likelihood is multiplied by priors and integrated over the nuisance parameters.

Input files for the BioEM analysis

In the following table, the BioEM integration ranges, priors, and input parameters (see details in https://github.com/bio-phys/BioEM) are presented for the first BioEM round of the systems studied.

BioEM parameter input for each system				
System	SemiSWEET	Hsp90	VGVAPG	TMEM16F
ORIENTATIONS	USE_QUATERNIONS	USE_QUATERNIONS	USE_QUATERNIONS	USE_QUATERNIONS
NUMBER_PIXELS	128×128	128×128	128×128	256×256
PIXEL_SIZE	0.7Å	2.2Å	0.3Å	1.059Å
CTF_DEFOCUS	[0.5,3.0] — 20gp	[0.5, 3.0] — 20gp	[0.1, 1.1] — 10gp	[1.0, 3.0] — 20 gp
CTF_B_ENV	[1, 1] — 1gp	[1, 1] — 1gp	[1, 1] — 1gp	[0.1, 0.1] — 1gp
CTF_AMPLITUDE	[0.1, 0.1] — 1gp	[0.1, 0.1] — 1gp	[0.1, 0.1] — 1gp	[0.1, 0.1] — 1gp
PRIOR_DEFOCUS_CENTER	1.75	1.75	0.6	1.75
SIGMA_PRIOR_DEFOCUS	4.0	4.0	1.7	3.0
SIGMA_PRIOR_B_CTF	1	1	1	3.0
DISPLACE_CENTER	[-2,2]	[-2,2]	[-1,1]	[-30,30]

Table 1. Parameters, integration ranges, and prior information for calculating the first BioEM round. The BioEM software was used to calculate probability $p(w_i|x_m)$ (see the Methods). The second BioEM round had a Gaussian prior for the CTF defocus centered around the experimental/synthetic defocus, and used a finer orientation grid around the best orientations from the first round. Abbreviation: gp= grid points. The center displacement was performed every pixel. See https://github.com/bio-phys/BioEM for details about the BioEM input keywords.

References

1. Branduardi, D., Gervasio, F. L. & Parrinello, M. From A to B in free energy space. *J. Chem. Phys.* **126**, 054103 (2007). DOI 10.1063/1.2432340.
2. Latorraca, N. R. *et al.* Mechanism of Substrate Translocation in an Alternating Access Transporter. *Cell* **169**, 96–107.e12 (2017). DOI 10.1016/j.cell.2017.03.010.
3. Abraham, M. J. *et al.* GROMACS: High performance molecular simulations through multi-level parallelism from laptops to supercomputers. *SoftwareX* **1-2**, 19–25 (2015). DOI 10.1016/j.softx.2015.06.001.
4. Cossio, P. & Hummer, G. Bayesian analysis of individual electron microscopy images: Towards structures of dynamic and heterogeneous biomolecular assemblies. *J. Struct. Biol.* **184**, 427–437 (2013). DOI 10.1016/j.jsb.2013.10.006.
