# Supplementary figures and images for: A Bayesian approach to extracting free-energy profiles from cryo-electron microscopy experiments
Source: Sci Rep. 2021 Jul 1;11:13657. doi: 10.1038/s41598-021-92621-1 (PMC8249403; doi:10.1038/s41598-021-92621-1)

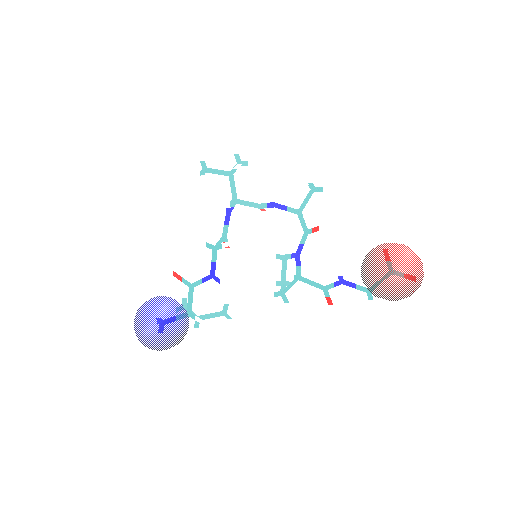

Supplement: Supplementary file 2 — Supplementary Video 1. [file 41598_2021_92621_MOESM2_ESM.gif]
